# Acupuncture as a Complementary Therapy in Conservative and Endodontic Procedures: A Narrative Review

**DOI:** 10.7759/cureus.75768

**Published:** 2024-12-15

**Authors:** Priyanka P, Shobana Krishnakumar, Mitthra Suresh, Lavanya Sacrapani, Vijay Venkatesh

**Affiliations:** 1 Conservative Dentistry and Endodontics, SRM Kattankulathur Dental College and Hospital, SRM Institute of Science and Technology (SRMIST), Chengalpattu, IND

**Keywords:** acupuncture, anxiety, endodontics, pain management, rootcanal

## Abstract

With over two millennia of history, traditional Chinese acupuncture ranks among the most renowned forms of supplementary and other healthcare. The use of acupuncture releases endorphins and serotonin, two naturally occurring painkillers, into the nervous system and stimulates it. It also modifies how pain signals are processed and perceived. The effectiveness of acupuncture in treating a range of dental issues has been demonstrated. Therefore, it is critical that dental professionals understand how acupuncture can be used to treat dental disorders. Painful endodontic conditions such as irreversible pulpitis and apical periodontitis often require root canal treatment (RCT), which is linked to a significant rate of postoperative pain. Short, acute pain from exposed dentin in reaction to stimuli is a defining feature of dentine hypersensitivity. The primary reason people seek urgent dental care is acute dental pain. However, alternative therapies, like acupuncture, are widely accepted as effective ways to manage dental pain in predental care. For this reason, pharmaceutical management works well, but it is often associated with side effects and occasionally may not offer sufficient pain relief. Acupuncture lessens anxiety related to dental procedures and reduces the need for analgesics following endodontic procedures, which may have unintended side effects.

## Introduction and background

Puncture using a needle is known as acupuncture. Acus, which means needle, and puncture, which means insertion, are the two Latin words that make up the term acupuncture [1]. According to traditional Chinese medicine (TCM), acupuncture is defined as the stimulation of particular points on or near the surface of the human body by any method of point stimulation, with or without the insertion of needles. These techniques include cupping, moxibustion (burning selected herbs on or over the skin), and electrical, magnetic, light, and sound energy, which are used to cure different medical diseases and to restore the normal physiological processes in humans. The Chinese theory holds that the human body is composed of yin and yang, two basic opposing life forces which are delicately balanced. The human body has two distinct circulation systems: yang (Qi) and yin (blood) [2]. Qi is also known as chi or energy (flows in a "jin-luo" or meridian), and yin flows inside the blood vessels [3]. Diseases related to a specific organ are due to obstructions in the flow of yang and yin [2]. It is, therefore, hypothesized that this blockage in the Qi's flow could be removed by inserting a needle. 

When a needle is inserted into an acupoint, neurotransmitters like histamine and bradykinin are released, causing a slight inflammatory reaction. The thick, myelinated A-delta fibers and the thin, unmyelinated C fibers, which are found in the skin and muscles, carry the stimuli to the central nervous system through synapses. The stimuli that terminate on the spinal cord's posterior horn cause enkephalinergic neurons to release enkephalin, a neurotransmitter that inhibits the perception of pain by blocking the substance P. The stimuli proceed primarily down the lateral spinothalamic tract until they reach the brainstem, where they increase cortisol secretion in the adrenal glands and release serotonin, which raises the endorphin and the adrenocorticotropic hormone (ACTH) levels which automatically affects the patient's stress and anxiety levels.

This led to the development of acupuncture's core therapeutic principle, which aims at removing the impediment to Qi flow. This established the basic therapeutic foundation of acupuncture. Consequently, acupuncture has gained popularity, especially in the treatment of pain [4], which includes dental analgesia. Yet, little is known about acupuncture's analgesic effects, which are frequently ascribed to its capacity to trigger the release of endorphins and serotonin from neurons and control how pain is perceived in the brain and spinal cord [5,6].

Microbial invasion of the dental pulp causes inflammation in the pulp and peri-radicular root tissues, resulting in acute pain [7]. It is believed that the root canal treatment (RCT), which is used to remove the tooth's intracanal infection, is an unpleasant operation. This belief might discourage people from accepting endodontic treatment, which would further rule out the prospect of preserving their natural teeth. The development of local anesthetics has resulted in the simple and predictable management of endodontic pain. However, certain conditions, like acute irreversible pulpitis, are obstacles to achieving profound anesthesia. Numerous factors, such as inflammation, anxiety, and anatomical limitations that the clinicians encounter, can contribute to these challenges. Postoperative discomfort after RCT is also rather common, with a prevalence as high as 58% [8,9].

Although the goal of both pharmaceutical and nonpharmacological methods is to reduce pain after nonsurgical RCT, these methods are not always effective and may even result in other unpleasant side effects. As already established, endodontic patients may benefit from acupuncture as a nonpharmacological alternative therapy both during and after surgery [10,11]. Additionally, in challenging circumstances, acupuncture may help reduce the need for analgesics during the postoperative period and supplement the local anesthetic in achieving the loss of sensation. Nonetheless, it is still not very common to use acupuncture in endodontic operations. This is probably because there isn't enough research assessing its ability to lessen endodontic discomfort [11].

## Review

Methodology

To conduct this review, a comprehensive search was undertaken using MEDLINE, PubMed, and Google Scholar. Keywords include “root canal,” “endodontics,” “root canal therapy,” “dental pain,” and "acupuncture in endodontics.” The search results were supplemented by reviewing the bibliographies of the articles found, ensuring a thorough exploration of the subject matter related to pain management, anesthesia, pulpitis, and acupuncture in conservative dentistry and endodontics. 

The clinical procedure of acupuncture

The following are the clinical steps for the administration of acupuncture before the commencement of dental treatment procedures:

Step 1

The current pain level experienced by the patient is recorded using the visual analog scale (VAS).

Step 2

The acupuncture point on the skin surface is sterilized with a cotton swab soaked in 70% alcohol. The most commonly used acupoints for dental analgesia include the large intestine four (LI4), the highest point of the adductor pollicis muscle which lies in the deepest muscular plane of the palm; the stomach 44 (ST44) located in between the second and third metatarsal joints on the midfoot; and the conception vessel 23 (CV23) located above the hyoid bone in the front of the neck, below the lower jaw, and above the thyroid cartilage.

Step 3

Separately packed disposable sterile Huanqiu stainless steel needles (Suzhou Huanqiu Acupuncture Medical Appliance Co., Ltd., China) of size 0.25 x 25 mm are inserted on the side of the painful tooth (perpendicular to the skin surface at points LI4 and ST44; parallel to the base of the tongue at CV23 acupoint). 

Step 4

After the needle insertion into the first acupoint on the same side of the tooth pain, the pain score is recorded again by the patient using the VAS score. This can be categorized as follows: if VAS = 0, no pain, the needle is removed, and the acupuncture procedure is terminated; if VAS > 0, associated with the decrease in pain or pain continued to exist, without disturbing the first needle, the second needle is inserted into the second acupoint, and the VAS pain score is recorded (during this procedure, acupuncture is terminated if VAS = 0; for VAS > 0, when the third needle is inserted without disturbing the first and the second, it ensures complete dental analgesia.

Step 5

All the needles are removed after five minutes, calculated from the time of insertion until VAS = 0; thus, the acupuncture procedure is terminated, and the dental treatment procedures are commenced accordingly [12].

Mechanism of action**

The basis for acupuncture's mechanism of action is the stimulation of muscular nerve endings via the needles inserted into particular energy meridians. This stimulation is then recognized and translated into three levels [12]. Hypothalamic level: when the hypothalamic-pituitary is activated, endorphins (which reduce pain), cortisol (which reduces inflammation), and serotonin (which reduces depression) are released into the bloodstream and the cerebrospinal fluid. Level of the midbrain: when grey matter neurons are activated, endorphins are released into the bloodstream and stimulate the production of serotonin and norepinephrine. At the level of the spinal cord, it leads to the release of dynorphins and the activation of interneurons in the gelatinous substance. 

When needles are used to activate nerve fibers like the A-delta and C fibers, pain conduction is inhibited. De qui, a sensation felt when the needle is inserted into the point, can appear as distention, pain, or numbness around the insertion site. This sets off a mild inflammatory reaction that releases bradykinin and histamine, among other neurotransmitters [13].

Dental disorders and acupuncture

In dentistry, acupuncture has the potential to treat a variety of conditions. For patients with conditions that traditional treatment modalities are unable to manage, acupuncture offers new hope. Acupuncture is effective for treating gag reflex and dental anxiety; dental pain, pain or disorders related to the temporomandibular joint (TMJ), or TMJ locks and clicks; xerostomia (dry mouth); atypical face discomfort; headache (tension headache, migraine); persistent muscle pain or spasm; neuropathic pain, particularly trigeminal neuralgia, or nerve pain; and anesthesia or paresthesia affecting the oral tissues. 

Review of clinical trials in conservative dentistry and endodontics

Acupuncture for Dental Pain

The dental pulp of the tooth and the periodontium around it contain a complex network of nerve fibers, and when stimuli activate these nerve endings, pain is immediately elicited. In order to manage dental pain, the etiology (such as periodontitis, caries, or gingivitis) needs to be determined and eradicated. If necessary, analgesic medications can be given orally.

Acupuncture's function in treating dental pain might not eradicate the source of the pain, but it assists in recovery. Promising results have been shown for postoperative dental pain, according to the November 1998 National Institutes of Health (NIH) Consensus Statement on acupuncture [14].

Ernst and Pittler [15] published a systematic review on the effectiveness of acupuncture in treating acute dental pain. Sixteen controlled trials were included in this review, the majority of which suggested that acupuncture was useful for dental analgesia. The reviewers concluded that dental pain could be reduced by acupuncture.

In contrast to these findings, a randomized controlled trial was carried out by Goddard and Albers [16] to investigate whether dull needling acupuncture at a specific acupoint could reduce the dental pulp sensory threshold caused by electrical pulp stimulation in the incisor teeth. Real or simulated 40 healthy adults who had never had acupuncture or had any incisor dental restorations received the latter form of treatment (with a dull needle that just contacted the skin, not penetrated it). There were no appreciable differences in pain relief between the volunteers who got the sham acupuncture and those who received the verum acupuncture. The researchers concluded that the dental pulp sensory threshold was not lowered by acupuncture. Chapman and associates [17]found that acupuncture significantly increased the tooth pain threshold compared to electrical stimulation. Short-term analgesia has also been demonstrated to be produced by acupuncture.

Dental pain may be reduced by acupuncture in the following ways [17]: inducing the production of endorphins and other neurohumoral substances (such as serotonin and neuropeptide Y) by stimulating the neurons in muscles and other tissues; modifying how the brain and spinal cord perceive and process pain; diminishing the anesthetic-induced cardiovascular response (linked to the adrenergic system); increasing adenosine release, which has antinociceptive characteristics; adjusting the limbic-paralimbic-neocortical network's activity; diminishing the inflammation by the facilitation of vascular and immunomodulatory factor release; and increasing microcirculation locally, which aids in the dispersal of edema [18].

Acupuncture for Dental Anesthesia

An additional method of achieving anesthesia during dental procedures is acupuncture. Prilocaine hydrochloride, a topical anesthetic, binds to sodium channels in the neuronal membrane, preventing sodium ions from entering. This inhibits the propagation of action potentials and nerve activity. The block is reversible, and nerve function recovers as the medication diffuses away from the cells. Research indicates that the onset of regional anesthesia following prilocaine hydrochloride administration is approximately two minutes. In 2003, Rosted and Bundgaard [19]conducted a study to detect if acupuncture might shorten the amount of time required for the onset of local anesthetic prior to the actual injection. Before receiving an injection of prilocaine hydrochloride for an inferior alveolar nerve block (IANB), the group that underwent local acupuncture had an induction time of 62 seconds, while the control group which only received the nerve block injection took approximately two minutes. The results of this study indicated that following the administration of a nerve block, regional acupuncture could hasten the induction period.

Reversal of dental analgesia by naloxone (AN) (opioid antagonist) using auricular electrical stimulation (AES) and dental pain threshold was assessed by Simmons and Oleson [20]. Forty participants were randomly assigned to one of four groups for which AES was followed by saline (AS), naloxone (AN), placebo AES followed by saline (PS), and placebo AES followed by naloxone (PN). A portable dental pulp tester was used to measure the dental pain threshold. The two placebo-stimulation groups (PS and PN) showed no variation, while the AES groups experienced a statistically significant 18% elevation. After receiving naloxone, the subjects in group AN experienced a decrease in their mean pain threshold, from <12% to over 23%. These results indicated that AES significantly raised pain thresholds, an effect that was partially counteracted by naloxone. This suggests that AES analgesia significantly increases the pain threshold, which is partially inhibited by naloxone. 

Acupuncture for Postoperative Dental Pain

Acupuncture's impact on postoperative pain following RCT in patients diagnosed with symptomatic apical periodontitis has been examined in one study. An equal number of subjects were randomly assigned to each of the two groups. Real acupuncture at the LI4 acupuncture point was administered to one of the groups, and mock acupuncture was administered to another. Fifteen minutes following the acupuncture session, each group underwent a single-visit randomized controlled trial. The acupuncture needle was taken out once the RCT was finished. Until the seventh postoperative day, patients were directed to document their level of pain following surgery using the VAS. Patients were prescribed 400 mg of ibuprofen as a rescue medication, and their analgesic consumption was also closely observed. When comparing the acupuncture group to the placebo group, a notable decrease in pain was seen. For instance, on the seventh postoperative day, the mean pain score for the acupuncture and placebo groups was 0.07 and 21.93, respectively. Moreover, the acupuncture group consumed significantly less analgesics [11].

Acupuncture for Irreversible Pulpitis

A single randomized controlled trial by Jalali et al. assessed the anesthetic efficacy of acupuncture in individuals suffering from irreversible pulpitis. Forty patients with irreversible pulpitis in the first or second mandibular molars were split into two groups. Acupuncture therapy was given at the LI4 point to one group, and sham acupuncture was given to the other. Following the administration of an IANB, RCT was carried out in both groups. The success rate of the IANB was contingent upon the additional need for supplemental anesthetic injection. The study's findings showed that IANB was substantially more successful in the acupuncture group, with 40% and 80% of patients in the acupuncture and control groups, respectively, needing additional injections [21].

Acupuncture for Pulpal Anesthesia

Gross et al. assessed the efficacy of acupuncture for pulpal anesthesia [2]. This clinical study included eight patients with 10 teeth that required vital pulp endodontic therapy. The first line of treatment for pain management was acupuncture. Six acupuncture points were used: gall bladder 7 (GB7) on both sides near the TMJ, gall bladder 44 (GB44) on both feet, and LI4 on both hands. One patient out of 10 reported complete analgesia, seven reported partial analgesia, and two reported no analgesia at all. Few acupoints used in dentistry are enumerated in Table 1 [22].

**Table 1:** Acupoints for dental conditions TMJ:  temporomandibular joints

Anatomical landmark	Name of acupuncture point	Associated recovery
Large intestine 4/Hegu (LI4)	Highest point of the adductor pollicis muscle	Facial pain and toothache
Stomach 44/Neiting (St44)	In-between the second and third metatarsal joints	Toothache, edema
Third-eye center/Yintang (M Hn3)	In-between the eyebrows	Anxiety and pain
Stomach meridian 6/Jaiche (ST6)	Angle of the mandible	Neuralgia and toothache
Stomach 7/Xiaguan (ST7)	Between the mandibular notch and zygomatic arch	Facial pain, toothache, and TMJ disorders
Conception vessel 23/Lianquan (CV23)	Above the hyoid bone	Facial pain
Gallbladder 44/Zuqiaoyin (GB44)	In-between the second and third toes	Chronic pain

Dental Anxiety and Gag Reflex

Ear acupuncture has been demonstrated in multiple controlled trials to be equally efficacious as intranasal midazolam in reducing gag reflex and dental anxiety. Karst conducted a randomized controlled trial and evaluated the use of auricular acupuncture to treat dental anxiety [23]. To lessen dental anxiety in patients having dental extraction, the efficacy of verum auricular acupuncture, intranasal midazolam, sham acupuncture, and no treatment at all was examined in this study. The groups that received midazolam and verum auricular acupuncture were considerably less anxious than the control and sham acupuncture groups. Additionally, the dentist found that intranasal midazolam and auricular acupuncture greatly increased patient compliance. The study's findings indicated that intranasal midazolam and auricular acupuncture were equally successful in treating dental anxiety.

Rosted and his colleagues [24] studied how acupuncture affected patients with high anxiety levels before receiving dental treatment. Regarding their methods for treating dental anxiety, eight dentists submitted 21 case reports. The Beck Anxiety Inventory (BAI) was used to measure anxiety levels. The BAI score was measured both prior to and following acupuncture treatment. Five minutes before the scheduled dental procedure, each patient had an acupuncture treatment. Following acupuncture treatment, the median value of BAI scores significantly decreased (from 26 points to 11 points with a probability value of p < 0.01), and in all 20 cases, the planned dental treatment could be completed.

A clinical study by Sari and Sari [25] examined the efficacy of acupuncture in treating gag reflex patients undergoing orthodontic treatment. The study looked into two acupuncture methods for treating gag reflex in orthodontic patients. Every patient had an upper dental alginate impression made, and the Gagging Severity Index (GSI) was used to gauge how severe their gag reflex was. Following acupuncture, the patient's gag reflex was assessed using the Gagging Prevention Index (GPI) with a second impression. When comparing the GPI values of the treatment groups to the GSI values of the placebo group, a significant decrease was noted. The researchers concluded that treating orthodontic patients' gag reflexes with acupuncture points was successful.

The antigagging point on the ear corresponds to the skin of the external auditory meatus (innervated by the auricular branch of the vagal nerve) and the skin adjacent to the auricle (innervated by the auriculotemporal branch of the mandibular division of the trigeminal nerve). The larynx, pharynx, and palatal region's sensory and motor functions are controlled by branches of the vagus nerve and the trigeminal nerves. One theory is that by stimulating the antigagging auricular acupuncture point, the gag reflex may be lessened by inhibiting muscular activity.

Acupuncture in Apical Periodontitis

Arslan et al. demonstrated that preoperative acupuncture treatment helped lower postoperative pain in teeth with symptomatic apical periodontitis in a randomized controlled trial that involved 30 participants [11]. Studies have shown that acupuncture may aid patients with symptomatic apical periodontitis and irreversible pulpitis in controlling their intraoperative and postoperative discomfort following RCT and reducing the failure rate of nerve blocks [21,26].

Acupuncture for Bruxism

The range of treatment options for bruxism is a result of its multifactorial etiology. It is acknowledged that occlusal, emotional, or both may be the cause of bruxism. Therefore, it is typically recommended to receive occlusal correction in addition to psychological treatment. The use of acupuncture in bruxism patients is based on the idea that it can lower masticatory muscle activity and manage stress and anxiety. The benefit of using acupuncture to treat bruxism is that there are no unfavorable side effects or potential complications.

Acupuncture in Xerostomia

Reduced or stopped salivary production due to a variety of systemic or local reasons is known as xerostomia. Since the 1980s, western medicine has recorded the use of acupuncture therapy as an alternative treatment for xerostomia. Acupuncture treatment may help pilocarpine-resistant xerostomia patients after radiation therapy for head and neck cancers, according to research by Johnstone [27] and Furness [28]. Although the exact method by which acupuncture can aid in increasing salivary flow is still unknown, several researchers have put up some potential theories, such as neuropeptides released during acupuncture therapy have trophic effects on the salivary gland acini and alter blood flow with anti-inflammatory qualities. Acupuncture therapy may use the neural circuit, which stimulates the salivary nuclei in the pons and, later, the salivary glands via the cranial nerves. Neural activations: activation of the parasympathetic nerves enhances salivary secretion.

Acupuncture in Neural Disorders

Facial palsy, trigeminal and other neuralgias, lower lip paresthesia or anesthesia after lower-third molar extraction, post-therapeutic neuralgias, and other neurological abnormalities can all be treated with acupuncture [29]. The TCM theory behind acupuncture for Bell's palsy is that by manipulating needles at both local and distal locations, Qi flow in the meridians can be controlled, Qi-blood equilibrium can be balanced, and the body's defenses against external wind pathogens can be strengthened. Additionally, acupuncture may aid in promoting nerve fiber regeneration and neural excitability. A connection between acupuncture and the autonomic nervous system has been suggested by certain studies [30,31]. The current definition of acupuncture as "acupuncture modulates the imbalance between the parasympathetic and sympathetic activity" may be consistent with TCM's belief that it restores the balance of Yin and Yang [32]. A point near the mandibular angle at the prominence of the masseter muscle and a point at the depression between the zygomatic arch and the mandibular notch are two examples of local acupuncture points used for facial palsy. The facial nerve's branches are found to be anatomically close to these two sites [33].

*Future Directions*

When administered by a qualified professional, acupuncture is regarded as a safe therapy because using traditional medications to treat acute dental pain can produce negative side effects like stomach ulcers and bleeding issues (ibuprofen), drowsiness, problems with stool, and insomnia (tramadol), as well as itching, skin rashes, and blood vessel disorders (paracetamol) [34].

Acupuncture is a natural and inexpensive therapeutic resource. Traditional surgical methods are not substituted by acupuncture [35]. Acupuncture is regarded useful as a symptomatic treatment for dental pain, according to recent research. When used on patients with pain who are waiting for dental care, it has a positive social impact and enhances the patient's physical and mental health, which helps the professional service succeed. The impact of acupuncture helps in lowering acute dental pain in patients who are waiting for treatment at emergency dental care centers after hours [12].

Acupuncture offers several benefits, including safety (as it is nontoxic with minimal side effects), in contrast to many other traditional treatment modalities; no dependence related to narcotics; easy and convenient if administered by a qualified practitioner [36]; no postoperative numbing effect; no nausea or vomiting; no potentiation of antihypertensive drugs; minimal postoperative pain and fewer side effects or serious complications; equipment is inexpensive; no interaction with any drug intake in patients taking medications for acute and chronic diseases; absence of dysgeusia and xerostomia; and complete safety in patients with autoimmune diseases and pregnancy. Additionally, because acupuncture doesn't damage the main blood vessels or nerves, it reduces the risk of iatrogenic injuries; hence, its clinical application during dental treatment is recommended [1]. 

*Limitations*

Acupuncture is linked to a variety of significant side effects that occur occasionally, such as internal organ injuries (like pneumothorax or cardiac tamponade) and infections like hepatitis C or HIV. Temporary side effects include the following such as needling pain or bleeding at the site of needling, which occurs in 7%-11% of all clinical cases [37].

Several large prospective studies have demonstrated that such side outcomes are quite rare, assuming acupuncture is performed by well-trained practitioners. An extensive research by Ernst and Sherman reported that about 2.4% of the adverse effects were recorded per 10,000 patients among 190,924 chronic pain sufferers. About 5% of the average death rate in the German population was reported [37]. A recent investigation in the United Kingdom found that nonmedically qualified acupuncturists interfere with their patients' prescription medications in 3% of cases, thereby posing an indirect danger to acupuncture use. Nonetheless, the evidence suggests that acupuncture, when performed by well-trained experts, is a relatively safe therapy. Serious side effects may result from the inadequate training experience of the huge number of paramedics using the approach [38].

Acupuncture also has several limitations which include the following: more time-consuming; failure to produce complete analgesia; it is considered ​inappropriate for use on children (due to the low tolerance for needles), and it is found to be ineffective when applied to needle-phobic patients (due to associated conditions such as ​lengthy induction time, bleeding at the site of needle insertion and unresponsiveness). Moreover, less research and less scientific data are currently available on the clinical efficacy of acupuncture for dental pain management in restorative and endodontic procedures. 

## Conclusions

Acupuncture is a useful traditional technique that can be applied in dentistry. Numerous studies have demonstrated the effectiveness of acupuncture in treating a variety of head and neck problems, particularly chronic illnesses such as myofacial discomfort and TMJ pain. Acupuncture may produce analgesic, antianxiety, and other therapeutic effects by mediating the intrinsic neural circuit, which plays a central role in the cognitive dimensions of pain.

In general, acupuncture should be viewed as a supplement to the conventional treatment. Its efficacy as a solitary analgesic for operational intervention is debatable; nonetheless, in the control of postoperative pain and the management of TMD discomfort, it may be a beneficial addition to the general dental practitioner's therapeutic protocol. These talents can be learned through a short postgraduate training program. Contrary to the common opinion, traditional medicine is plagued by the unintended consequences of alternative therapies that are completely safe. However, some harmful effects of acupuncture include pneumothorax, endocarditis, and hepatitis, all of which can be fatal. It should be noted that the majority of these effects are caused due to the lack of basic anatomical knowledge or the failure to use standardized aseptic methods by nonmedical/nondental qualified practitioners. Acupuncture is still considered a clinically safe practice, in the hands of a professionally qualified practitioner.

Future research may provide a wide insight into the underlying processes for the dental applications of acupuncture in regular clinical practice, and when the principles of TCM are widely accepted, more large-scale experimental investigations with enhanced experimental designs are required to confirm acupuncture's uses in dentistry and other fields.
